# Metalware-associated orthopaedic infections caused by *Staphylococcus lugdunensis*: An emerging pathogen

**DOI:** 10.1016/j.jinf.2017.05.013

**Published:** 2017-10

**Authors:** Ang Li, Nicholas Gow, Bridget L. Atkins, Adrian Taylor, Tim Peto, Martin A. McNally, Philippa C. Matthews

**Affiliations:** Bone Infection Unit, Nuffield Orthopaedic Centre, Oxford University Hospitals NHS Foundation Trust, Windmill Road, Oxford, UK; Bone Infection Unit, Nuffield Orthopaedic Centre, Oxford University Hospitals NHS Foundation Trust, Windmill Road, Oxford, UK; Department of Infectious Diseases and Microbiology, Oxford University Hospitals NHS Foundation Trust, John Radcliffe Hospital, Headley Way, Oxford OX3 9DU, UK; Department of Infectious Diseases, North Shore Hospital, Auckland, New Zealand; Bone Infection Unit, Nuffield Orthopaedic Centre, Oxford University Hospitals NHS Foundation Trust, Windmill Road, Oxford, UK; Department of Infectious Diseases and Microbiology, Oxford University Hospitals NHS Foundation Trust, John Radcliffe Hospital, Headley Way, Oxford OX3 9DU, UK; Bone Infection Unit, Nuffield Orthopaedic Centre, Oxford University Hospitals NHS Foundation Trust, Windmill Road, Oxford, UK; Department of Infectious Diseases and Microbiology, Oxford University Hospitals NHS Foundation Trust, John Radcliffe Hospital, Headley Way, Oxford OX3 9DU, UK; Nuffield Department of Medicine, John Radcliffe Hospital, Headley Way, Oxford OX3 9DU, UK; Bone Infection Unit, Nuffield Orthopaedic Centre, Oxford University Hospitals NHS Foundation Trust, Windmill Road, Oxford, UK; Department of Infectious Diseases and Microbiology, Oxford University Hospitals NHS Foundation Trust, John Radcliffe Hospital, Headley Way, Oxford OX3 9DU, UK; Nuffield Department of Medicine, Peter Medawar Building for Pathogen Research, University of Oxford, South Parks Road, Oxford OX1 3SY, UK

**Keywords:** Prosthetic joint infection, Bone infection, Orthopaedic infection, *Staphylococcus aureus*, *Staphylococcus lugdunensis*, Epidemiology, Antibiotics, Management

*To the Editor*:

The increasing use of molecular diagnostics (e.g. MALDI-TOF) that facilitate speciation of coagulase negative staphylococci (CoNS), has led to enhanced detection of *Staphylococcus lugdunensis* as the causative agent of a variety of infections. A series of cases of infective endocarditis among patients with *S. lugdunensis* bacteraemia was recently reported in this journal by Non et al.^1^ In addition to other reports of endocarditis,^2^, ^3^, ^4^ this organism has also increasingly been recognized in association with invasive infections at other sites including those arising in an orthopaedic setting.^5^, ^6^ A recent retrospective study of prosthetic joint infections (PJI) in France found that *S. lugdunensis* may result in less favourable outcomes than other CoNS, with reported outcomes similar to *S. aureus*.^7^ The optimal management of *S. lugdunensis* orthopaedic infections is not established.

On this basis, we undertook a study to evaluate the contribution of *S. lugdunensis* to prosthetic orthopaedic infections, and to evaluate treatment and outcomes. We interrogated electronic records for patients treated under the care of Oxford trauma team and/or Bone Infection Unit, a specialist UK tertiary referral centre for bone and joint infection that handles approximately 200 revision arthroplasties each year. At this centre, patients are routinely started on broad-spectrum antimicrobials intraoperatively, after multiple samples are taken.^8^ Once identification and sensitivity testing are complete, antimicrobial therapy is adjusted according to susceptibility testing. Traditionally, most patients with confirmed deep bone and joint infection have been treated with six weeks of parenteral therapy before switching to oral antibiotics.

We identified cases of *S. lugdunensis* retrospectively, among patients age ≥18 years, by reviewing a 24 month period commencing April 2014 (when MALDI-TOF was first introduced into the diagnostic laboratory). We collated information pertaining to microbiology samples, surgical intervention, choice, route and duration of antibiotic therapy, and clinical outcomes. As a denominator group, we identified patients with *S. aureus* prosthetic hip and knee infections. Patient identifying information was removed before further analysis. As this was undertaken as a quality improvement project with no intervention, ethical approval was not required.

We identified 68 *S. lugdunensis* isolates from 20 unique patients (Suppl. data table 1). Median age was 69 years (IQR 40–86). Half of the patients were diagnosed with PJI, with knees outnumbering hips (n = 7 vs n = 3, respectively). Eight patients with *S. lugdunensis* were treated for infection of fracture fixation metalware and two for spinal metalware infection. A majority (17/20) of these patients had recorded surgical intervention; variable approaches were undertaken for PJI including debridement and implant retention (‘DAIR’), single stage revision and two stage revision. All of the fracture fixation devices were removed with excision of infected bone and reconstruction.

Over the same time frame, we identified 80 cases of knee and hip PJI caused by *S. aureus* (n = 45 vs n = 35, respectively; Suppl. data table 2). Although the distribution of *S. lugdunensis* between hip and knee PJI was not statistically different from that seen for *S. aureus* (p = 0.5, Fisher's Exact Test, Fig. 1A), an apparent enrichment of *S. lugdunensis* infections among prosthetic knees compared to hips is a feature that has been noted elsewhere.^9^ This is not as a result of a substantially greater number of knee than hip replacements (the UK National Joint Registry reports 796,636 hip and 871,472 knee replacements in England, Wales and Northern Ireland between April 2003 and December 2015^10^). There was no difference in age between patients with *S. lugdunensis* vs *S. aureus* PJI (median 69 years vs 70 years, respectively, p = 0.8, Fig. 1B).

**Figure 1:**
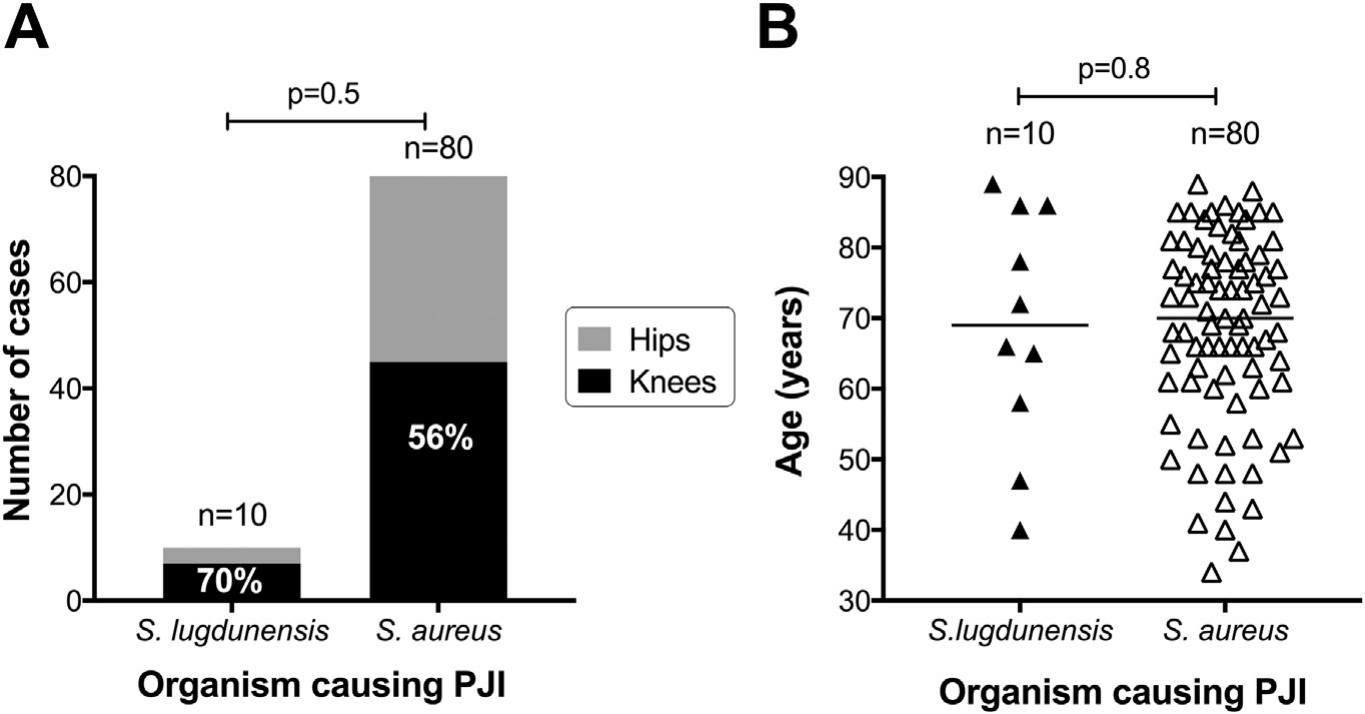
*S. lugdunensis* and *S. aureus* as a cause of knee and hip prosthetic joint infections (PJI) in a UK cohort over two years from April 2014. A: Absolute case numbers and relative contributions of the two organisms to knee and hip PJI. Percentages shown on black bars indicate proportion of all cases that are associated with knee infection. P-value by Fisher's Exact Test. B: Age distribution of patients with *S. lugdunensis* and *S. aureus*. Horizontal lines indicate median. P-value by Mann–Whitney test.

None of the 68 *S. lugdunensis* isolates demonstrated resistance to glycopeptides, daptomycin, oxacillin, rifampicin, clindamycin or linezolid, but 14 isolates from 4 different patients were found to demonstrate *in vitro* resistance to at least one antistaphylococcal agent (co-trimoxazole, tetracycline, fucidic acid, gentamicin); Suppl. data table 3.

Details of antibiotic therapy are shown in Suppl. data table 1. Following the immediate postoperative period, nine patients continued with intravenous therapy for a median of six weeks (glycopeptide or ceftriaxone, at the discretion of the infectious diseases/microbiology consultant). A variety of oral treatment strategies were employed, with the most common being dual therapy with ciprofloxacin and rifampicin (n = 8). Therapy duration ranged from 6 to 21 weeks for 15 patients, whilst three underwent a strategy of longer term suppressive therapy on the grounds that infected metalware could not be removed or adequately debrided. We were unable to determine the antibiotic strategy retrospectively for two patients.

Follow up data were available for 16 patients (at median 118 days following operation/initiation of antimicrobials, range 43–583 days), of whom 15 (94%) had no reported evidence of ongoing or recurrent infection. One patient was found to have a minimally symptomatic persistent fracture non-union and a decision was made to continue with a suppressive strategy, without further surgery. Outcome data were unavailable for the remaining four patients, probably due to transfer out of area. There was no difference between infection outcomes for patients receiving intravenous followed by oral antimicrobial strategies, when compared to those treated with oral antimicrobials alone, although a substantially larger prospective study is required to determine whether oral therapy is non-inferior. The active approach to thorough surgical debridement in the majority of cases, together with the susceptibility data of *S. lugdunensis* is in keeping with the good clinical outcomes.

In conclusion, this small series confirms a niche for *S. lugdunensis* as a cause of orthopaedic infections involving prosthetic material. In knee and hip PJI in our centre, it accounts for cases at a ratio of 1:8 compared with *S. aureus*. Ascertainment of cases is likely to continue while molecular approaches to diagnostics become more widely adopted.

Optimum therapy has not been clearly defined, and we have demonstrated variation in antimicrobial prescribing practice. Given the small numbers of patients, it is not currently possible to advocate any particular management approach to *S. lugdunensis* prosthesis infections. However, *in vitro* data confirm an organism that is susceptible to conventional therapy for *S. aureus*, and outcomes appear to be good when patients are treated using surgical and medical management protocols equivalent to those employed for *S. aureus*. Further work is needed to establish with more confidence the optimum route, choice and duration of antimicrobial therapy in order to unify a treatment approach.

## Authors' contributions

AL and PM conceived and designed the study. AL, NG, TP and PM collected and analysed the data. BA, AT and MM provided advice on analysis and manuscript drafting. AL and PM wrote the manuscript. All authors have seen and approved the final version.

## Competing interests

The authors declare that they have no competing interests.

## Appendix A Supplementary data

Suppl. data table 1. Characteristics of patients with *Staphylococcus lugdunensis* orthopaedic infections associated with prosthetic material identified in the two year period beginning April 2014.

Suppl. data table 2. Patients with *Staphylococcus aureus* prosthetic knee and hip joint infections identified in the two year period beginning April 2014. MSSA = methicillin susceptible *S. aureus*; MRSA = methicillin resistant *S. aureus*.

Suppl. data table 3. *In vitro* susceptibility testing for 67 isolates of *Staphylococcus lugdunensis* from 20 patients with orthopaedic infections associated with prosthetic material. MIC for each organism was ascertained by BD Phoenix; breakpoints are given in mg/L based on EUCAST data (http://www.eucast.org/clinical_breakpoints). Nd = no data.
